# Two-year outcomes of intravitreal aflibercept in a Swiss routine treat and extend regimen for patients with neovascular age-related macular degeneration

**DOI:** 10.1038/s41598-020-76354-1

**Published:** 2020-11-20

**Authors:** Andreas Ebneter, Stephan Michels, Christian Pruente, Pascal Imesch, Felix Eilenberger, Susanne Oesch, Isabelle P. Thomet-Hunziker, Katja Hatz

**Affiliations:** 1 Department of Ophthalmology and Department for BioMedical Research, Inselspital, Bern University Hospital, University of Bern, Bern, Switzerland; 2 Augenklinik Zürich West, Zurich, Switzerland; 3 University of Zürich, Zurich, Switzerland; 4 Department of Ophthalmology, University of Basel, Augenklinik, Basel, Switzerland; 5 Institute of Molecular and Clinical Ophthalmology Basel (IOB), Basel, Switzerland; 6 EYEPARC, Bern, Switzerland; 7 Alcedis GmbH, Giessen, Germany; 8 Bayer (Schweiz) AG, Zurich, Switzerland; 9 Vista Klinik, Binningen, Switzerland; 10 Faculty of Medicine, University of Basel, Basel, Switzerland

**Keywords:** Outcomes research, Macular degeneration

## Abstract

The aim of this observational study was to assess the use and outcome of intravitreal aflibercept in a treat and extend regimen in treatment-naïve neovascular AMD patients in routine practice. This both retrospective and prospective study was conducted in four larger Swiss retina clinics (ASTERIA study). The primary endpoint was the mean change in best-corrected visual acuity (BCVA) in ETDRS letters from baseline to 12 months. Between December 2017 and August 2018, 160 patients were included. For patients with available data, the mean change in BCVA was + 8.4 (± 14.4) letters at month 12 (n = 139) and + 5.0 (± 11.4) letters at month 24 (n = 95). A mean number of 8.3 (± 2.4) injections were administered within the first year and 5.4 (± 2.9) injections during the second year. On average, the observed treatment interval at month 12 was 63.3 (± 22.0) days and increased to 69.1 (± 28.6) days at month 24. For 37% of the patients, a treatment interval ≥ 12 weeks was attained at month 24. In conclusion,  intravitreal aflibercept in a Swiss real-life treat and extend regimen resulted in comparable anatomic and functional outcomes as were observed in the prospective registration trials of aflibercept for nAMD treatment.

## Introduction

When VEGF inhibitors were first registered by health authorities for the treatment of neovascular age-related macular degeneration (nAMD), the approved dosing regimens involved fixed monthly intravitreal injections which proved to be ground-breaking at slowing progression of disease and improving vision in pivotal clinical trials^1–3^. However, due to the treatment burden and economic limitations in clinical practice, anti-VEGF agents were subsequently more frequently used pro re nata (PRN), a reactive approach, where patients are monitored monthly but treated only when functional and/or anatomical parameters have worsened. Because of inherent systematic undertreatment and frequent reactivation, PRN treatment leads to suboptimal functional outcomes as reported in many real-life analyses in different countries^4^. Moreover, a substantial disadvantage of PRN regimens is the need for frequent follow-up visits, commonly every 4 weeks. Mantel and co-workers have found that nAMD patients have their intrinsic individual treatment interval, ranging from 4 to 14 weeks which is relatively stable over time in most of them^5^. Similar results were shown for intraocular VEGF level rebounds after ranibizumab or aflibercept treatment, however, indicating longer VEGF suppression with aflibercept^6^. This goes along with a longer intravitreal half-life for aflibercept compared with other anti-VEGF drugs and theoretically a longer biologic activity in the eye^7,8^.

The suboptimal functional gains seen with PRN regimens prompted the need for a new treatment regimen with individualised follow-up intervals and better outcomes. The proactive “treat and extend” (T&E) treatment approach met these requirements best. It delivered better visual results, presumably because by design this regimen aims at proactively maintaining a dry or at least stable macula, which results in fewer relapses and less irreversible structural damage and scarring. This treatment concept was first proposed by Spaide^9^ and shown to be effective in the LUCAS study, the first prospective clinical trial evaluating T&E for ranibizumab and bevacizumab^10^. The advantages of T&E regimens were promptly recognised and respective treatment was adopted early by many clinical sites in Switzerland. This led to registration with a different treatment label for intravitreal aflibercept (IVT-AFL) in Switzerland compared with the US and the rest of Europe. It allowed for a T&E regimen of IVT-AFL already in the first year of treatment and furthermore gave the option of treating as frequently as every 4 weeks, if needed. This current study was initiated to gain insight into the outcome and manageability of T&E IVT-AFL in treatment-naïve patients with nAMD in routine clinical practice at larger Swiss retina clinics.

## Materials and methods

### Study design

ASTERIA was a multi-centre, observational study conducted at four ophthalmic clinics in Switzerland. All patients provided written informed consent and were enrolled between December 2017 and August 2018. Study data were documented retro- and prospectively for up to 24 months after IVT-AFL treatment initiation using electronic case report forms.

### Ethics approval

Ethical approval of the study was obtained via a centralized process at the Ethics Committee for north-west and central Switzerland (Project-ID: 2017-01864) which also applies to all other involved Ethics Committees. The study was conducted in accordance with all applicable laws and regulations, ICH-GCP guidelines were followed whenever applicable. The study was registered at ClinicalTrials.gov on December 11, 2017 with the clinical trial registration number NCT03382587.

### Patients

Male and female patients more than 55 years of age with treatment-naïve neovascular AMD for whom the decision to initiate treatment with IVT-AFL in a T&E regimen had been made according to routine clinical practice at the site of enrolment. Exclusion criteria were contraindications as listed in the local Summary of Product Characteristics (e.g. ocular or periocular infection and active or suspected intraocular inflammation). Patients with other eye diseases (e.g. advanced glaucoma or visually significant cataracts) likely to require surgery in the study eye, as well as patients with concomitant ocular or systemic administration of drugs within 3 months prior to IVT-AFL initiation that could potentially interfere with or potentiate the mechanism of action of IVT-AFL were also excluded. Only the eye with longer IVT-AFL treatment history was considered for inclusion in the study in retrospectively documented patients in whom both eyes fulfilled eligibility criteria.

### Treatment

Within the scope of the study, the T&E regimen was generally defined as follows: (1) initial doses every 4 weeks until a dry retina or disease stability was reached; (2) afterwards, treatment intervals were extended if disease stability was maintained and no signs of worsening, e.g. new or recurrence of exudation (as measured by optical coherence tomography), haemorrhage, or visual acuity reduction were observed on the injection day; (3) if signs of recurrence were observed at the injection day, the subsequent treatment interval was shortened. The extension and shortening increments were not mandated by the protocol but were at the discretion of the treating physician according to local clinical routine and varied typically between one and two weeks.

All decisions pertaining to diagnostic procedures and treatments including the decision to initiate IVT-AFL using T&E regimen were made by the treating physician according to routine clinical practice and independently of study participation. Therefore, planning and timing of visits and injections varied according to the patient’s need and the standards adopted by study centres.

### Objectives

The primary endpoint of this study was the mean change in best-corrected visual acuity (BCVA) from baseline (i.e. initiation of IVT-AFL) to month 12. BCVA was assessed in accordance with each institution's routine clinical practice either by using the Snellen or the Early Treatment Diabetic Retinopathy Study (ETDRS) charts. Further key outcomes included the mean change in BCVA from baseline to month 24, the mean change in central retinal thickness (CRT) as determined by spectral domain optical coherence tomography from baseline to months 3, 12, and 24, the mean number of injections and the mean treatment intervals at months 12 and 24, new or increasing disease activity indicated by haemorrhage, increasing pigment epithelial detachment (PED), intraretinal or subretinal fluid, and retinal pigment epithelial (RPE) rip within 12 and 24 months after IVT-AFL initiation. Additionally, safety was analysed.

### Statistical analysis

All statistics were exploratory or descriptive and were performed using the SAS software version 9.4 (SAS Institute, Cary, USA). Two analysis subsets were defined for statistical analysis: All patients with at least one documented IVT-AFL administration were included in the safety set (SAF set). Patients included in the safety set for whom BCVA assessment for the study eye was available at least for baseline and one further visit were included in the effectiveness set (EFF set). To account for different notations of BCVA (ETDRS or Snellen charts), Snellen values were converted to ETDRS letters for analysis according to Gregori et al.^11^. The analyses were performed based on the observed data at month 3 (90 ± 21 days), month 12 (360 ± 49 days), and month 24 (720 ± 56 days). The Spearman rank correlation was used to analyse the change in BCVA (observed cases) at 12 and 24 months in relation to the number of applied IVT-AFL injections. The mean change in BCVA and CRT between baseline and the respective timepoints for the total population were checked for significance using the paired t-test in a post hoc analysis. To impute missing post-baseline data, the last observation carried forward (LOCF) approach was used for analysis of endpoints evaluated at a specific time point (e.g. change in BCVA). Statistical subgroup analyses of the EFF set were additionally performed according to the type of choroidal neovascularisation (CNV) at baseline. Prism software (GraphPad Software Inc. version 7.05, San Diego, CA, USA) was used for data presentation.

## Results

### Patient disposition and baseline characteristics

Between December 2017 and August 2018, 163 patients were enrolled. Three patients in retrospect did not fulfil eligibility criteria and were excluded from the analysis (Supplementary Fig. S1). In total, 160 patients were included in the SAF set. All patients were included retrospectively, hereof 20 patients had a partly prospective therapy documentation. For all patients, BCVA values were available at baseline and at one subsequent visit at least, such that all 160 patients were included in the EFF set which is therefore identical to the SAF set (see Supplementary Fig. S1). In total, 101 patients (63%) completed the study (24 months ± 8 weeks) and 59 patients (37%) discontinued study participation prematurely. Of these, 19 patients successfully stopped therapy with IVT-AFL prior to month 24 due to the absence of disease activity, and another 12 patients stopped IVT-AFL treatment because of insufficient response. Besides, 10 patients changed dosing regimen (mainly to PRN), 8 patients had an interfering ophthalmic treatment (e.g. cataract surgery, photodynamic therapy), 4 patients were lost to follow-up, 2 patients discontinued due to adverse events, and 4 patients for other reasons (systemic disease or patient´s wish). Demographic data and disease characteristics for the patients in the EFF set are shown in Table 1.

**Table 1:** Patient demographics and disease characteristics at baseline (prior to initiation of IVT-AFL).

Demographic parameter/disease characteristic	EFF Set (n = 160)
Age at baseline [years]	
Median (min, max)	81 (58, 97)
Mean (SD)	80.9 (8.3)
Gender	**n (%)**
Female	89 (55.6)
Male	71 (44.4)
Type of choroidal neovascularisation at baseline	**n (%)**
Occult	102 (63.8)
Predominantly classic	27 (16.9)
Polypoidal choroidal vasculopathy	13 (8.1)
Minimally classic	9 (5.6)
Retinal angiomatous proliferation	3 (1.9)
Missing	6 (3.8)
Disease features at baseline	**n (%)**
Haemorrhage	41 (25.6)
Pigment epithelial detachment	116 (72.5)
Subretinal fluid	125 (78.1)
Cystoid intraretinal fluid	92 (57.5)
RPE rip	3 (1.9)
Baseline BCVA	
ETDRS letter score, Mean (SD)[letters]	61.5 (20.1)
Approximate Snellen equivalent [ft]	20/125
Retinal thickness at baseline [µm]	
Mean (SD)	404.2 (134.5)

### Change in BCVA

**Figure 1:**
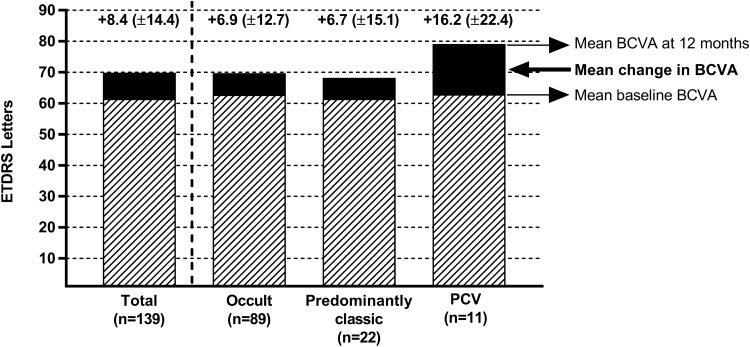
Mean change (± standard deviation) in best-corrected visual acuity (BCVA) at month 12 based on observed data for the total cohort and according to the three most common choroidal neovascularisation (CNV) types at baseline. PCV: polypoidal choroidal vasculopathy.

Baseline BCVA for patients in the EFF set with available BCVA data at month 12 (n = 139) was 61.5 (± 19.7) ETDRS letters (Snellen equivalent 20/125). For these patients, BCVA had significantly increased by 8.4 (± 14.4) letters at month 12 compared to baseline (95% CI 6.0–10.9, *p* < 0.01) (Fig. 1). The mean change in BCVA at month 12 differed nominally between the CNV type subgroups and was particularly high in patients with polypoidal choroidal vasculopathy (16.2 (± 22.4) letters; Fig. 1). When using the LOCF approach, the mean change from baseline at month 12 was 8.3 (± 14.6) letters for the total EFF set (n = 160). At 24 months after IVT-AFL initiation, the mean change in BCVA compared to baseline was + 5.0 (± 11.4) letters (95% CI 2.6–7.3, *p* < 0.01) for the total EFF set (n = 95 observed cases). The LOCF approach yielded a mean BCVA change of 6.6 (± 15.4) letters at 24 months.

At months 12, 30.6% (n = 49) of the patients had gained, whereas 4.4% (n = 7) had lost 15 or more letters compared to baseline. Even 24 months after IVT-AFL initiation, 25.6% of patients (n = 41) had an increase in BCVA of 15 or more letters, and for 6.7% (n = 11) BCVA had deteriorated by 15 or more letters compared to baseline.

### Change in CRT

**Figure 2:**
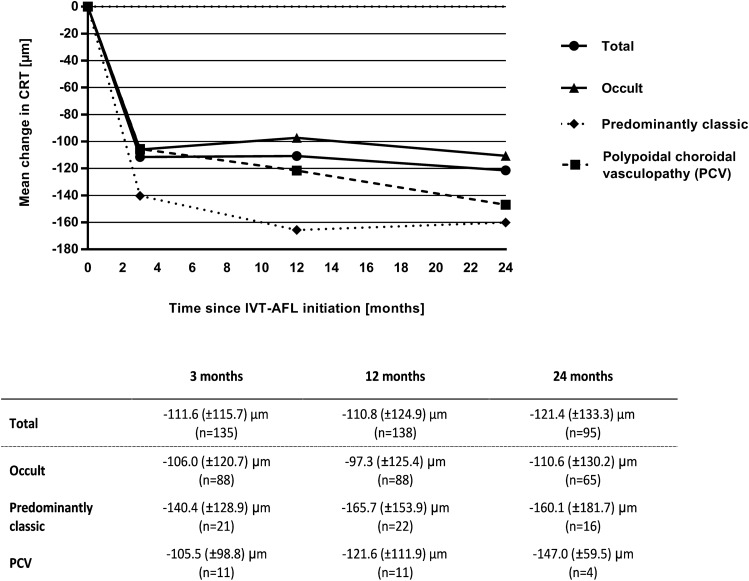
Mean change in central retinal thickness (CRT) over time based on observed cases for the total cohort and according to the main choroidal neovascularisation (CNV) types at baseline.

Starting with a mean CRT of 404.2 (± 134.5) µm, the mean change in CRT compared to baseline for the EFF set was − 111.6 (± 115.7) µm (95% CI − 131.3–− 91.9, *p* < 0.01) 3 months after IVT-AFL initiation, which remained stable at 12 months (− 110.8 (± 124.9) µm; 95% CI − 131.8–− 89.8, *p* < 0.01) and at 24 months (− 124.4 (± 133.3) µm; 95% CI − 148.5–− 94.3, *p* < 0.01). Also, for the three main CNV type subgroups, the CRT was nominally clearly and sustainably reduced (Fig. 2) with IVT-AFL treatment. The CRT reduction was particularly marked for the predominantly classic subtype.

### Number of injections and treatment intervals

Patients in the EFF set received a mean of 8.3 (± 2.4) IVT-AFL injections in the study eye in the first year and 5.4 (± 2.9) injections in the second year (see Supplementary Fig. S2 including also the number of injections of the three main CNV type subgroups). Spearman analysis indicated no correlation between the number of IVT-AFL injections and the mean change in BCVA (observed cases) at 12 months (Spearman coefficient − 0.12; *p* = 0.1740) or at 24 months (Spearman coefficient − 0.06; *p* = 0.5642).

**Figure 3:**
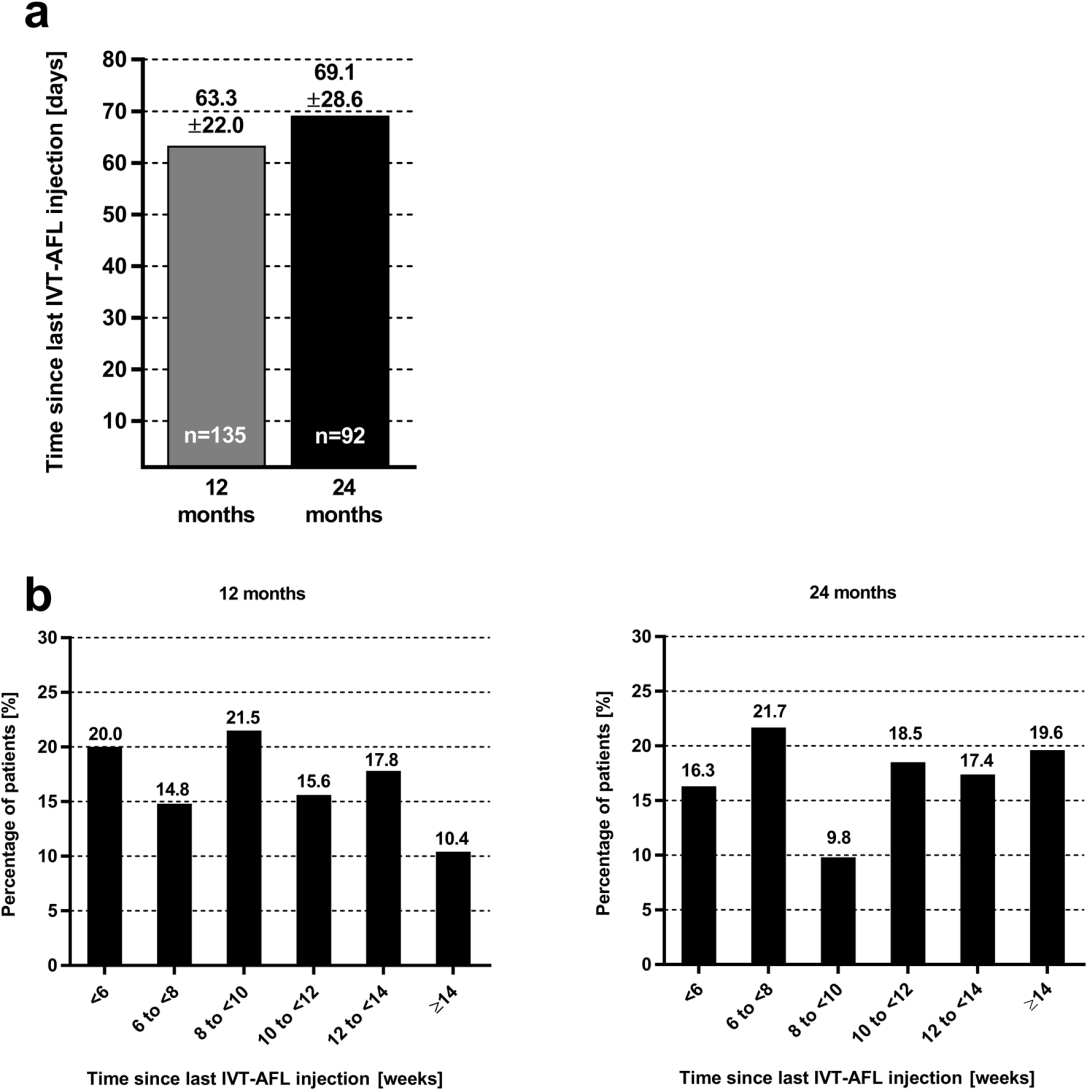
Mean time since last intravitreal aflibercept (IVT-AFL) injection at 12 and at 24 months after IVT-AFL initiation analysed as continuous (**a**) and as categorical (**b**) variable based on observed cases.

To evaluate the treatment intervals during the T&E IVT-AFL regimen, the time since the last injection at 12 and 24 months was analysed. As shown in Fig. 3a, the mean time since last injection for the total EFF set increased from 63.3 (± 22.0) days at month 12 to 69.1 (± 28.1) days at month 24. For 37.0% of patients, the time since last injection at month 24 was 12 weeks or more. However, for some patients the time since last injection was still < 8 weeks at month 12 (35% of patients) and at month 24 (38% of patients) (Fig. 3b). When considering only treatment intervals without any new disease activity between two injections (maintained intervals), the last maintained intervals (SD) before 12 months (61.4 ± 22.5 days) and before 24 months (65.9 ± 28.1 days) were only slightly different.

### Disease activity

In the 160 patients of the EFF set, new or increasing disease activity occurred in 77 patients (48.1%) within 12 months and in 91 patients (56.9%) within 24 months from baseline. For most of the patients with new or increasing disease activity during the study, the occurrences were only mild (79 patients) whereas the other patients (n = 12) had at least one severe occurrence as classified by the investigator.

**Figure 4:**
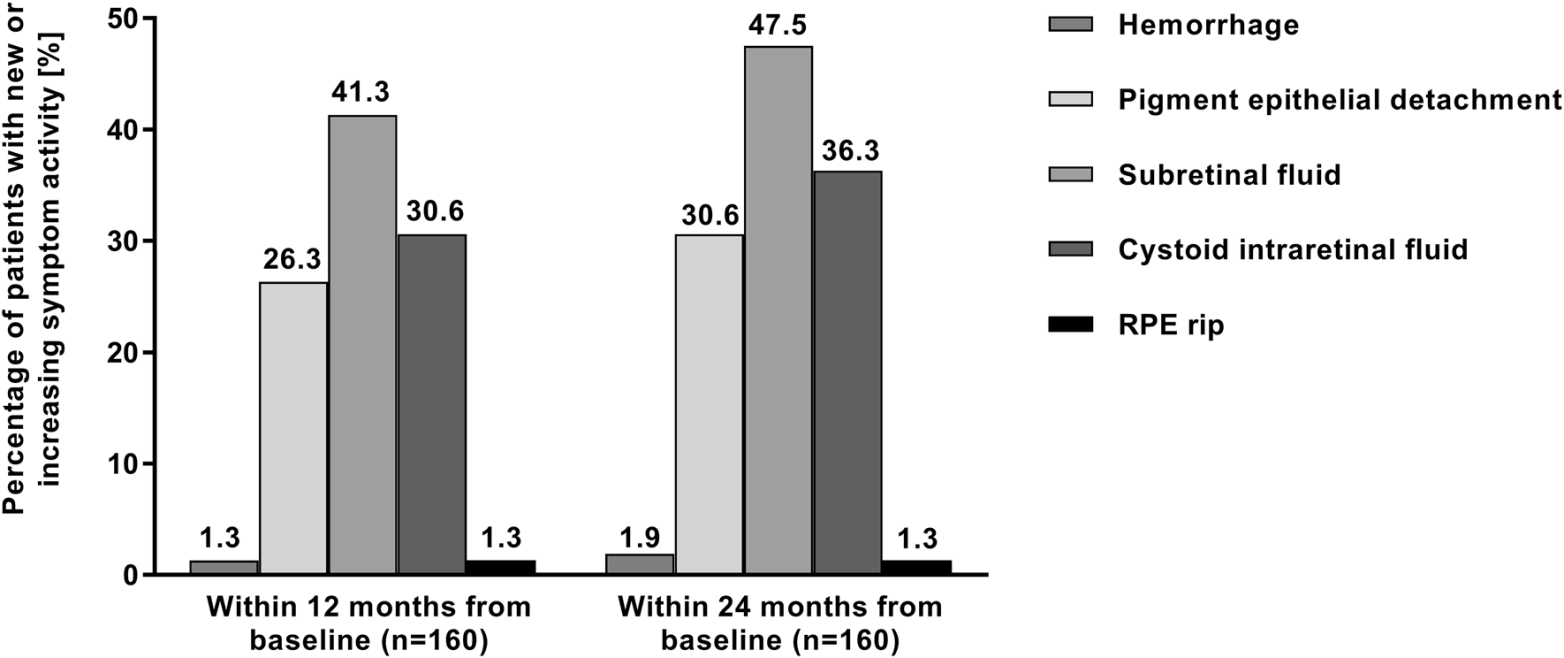
Features of new or increasing activity within 12 and 24 months after intravitreal aflibercept (IVT-AFL) initiation. RPE: retinal pigment epithelium.

Moreover, features associated with disease activity of specific interest were assessed in more detail. The frequency of occurrence of the most important features found in eyes with new or increasing disease activity are shown in Fig. 4.

### Safety analysis

The safety set comprised the 160 patients with at least one IVT-AFL injection. Treatment-emergent adverse events (TEAEs) have occurred in 17 (10.6%) patients (see Supplementary Table S1). In three patients (1.9%) non-ocular TEAEs and in 14 patients (8.8%) ocular TEAEs were reported. Most commonly reported ocular TEAEs were nAMD in the fellow eye (5 patients (3.1%)), cataract operation (4 patients (2.5%)), and corneal erosion (2 patients (1.3%)). No cases of endophthalmitis or intraocular inflammation were reported during the study.

## Discussion

In recent years, T&E regimens have been increasingly used in routine clinical practice for patients with nAMD treated with anti-VEGF therapy. T&E approaches have been conceived to not only reduce the treatment and monitoring burden compared with PRN treatment, but also to gradually adapt the follow-up intervals to the individual needs. The proactive approach should also minimise the risk of severe recurrences of nAMD. Furthermore, with T&E regimens the patient´s uncertainty about examinations and procedures during the visit is minimal, since an intravitreal injection is given at each scheduled encounter.

Within this observational study, the outcome of patients treated with IVT-AFL administered in a T&E regimen for 24 months in Swiss routine clinical practice was assessed. The patients’ mean baseline BCVA (61.5 (± 20.1) ETDRS letters) was higher than that observed in other interventional^12^ and non-interventional studies^13,14^ indicating that in Switzerland patients with nAMD are diagnosed early and rapidly treated. Despite the high baseline BCVA with potential ceiling effect, vision increased significantly by a mean of 8.4 (± 14.4) ETDRS letters at 12 months. This is a considerably better outcome than that reported in another observational Swiss T&E IVT-AFL study with only a 5.7 letter improvement^15^. The visual outcome in the current study is in line with the results of the interventional T&E IVT-AFL ATLAS study^12^ and closely matches the interventional VIEW trials that involved fixed, bimonthly IVT-AFL administration^16^ and reported vision gain of 8.4 letters in the q8 group after 12 months. After 24 months, a significant vision gain of 5.0 (± 11.4) ETDRS letters was observed in our study. The BCVA change with LOCF at 24 months was 6.6 (± 15.4) letters. The better BCVA gain in the LOCF analysis might have been influenced by patients who left the study early due to treatment success and potentially good visual outcomes. However, the further BCVA development of these patients was not recorded anymore in this study. Our 24 months BCVA results are in line with the 6.0 letters found by the Fight Retinal Blindness Study Group^17^ and the 7.0 letters reported by Jaki Mekjavić et al.^18^, both having analysed T&E IVT-AFL under routine conditions.

During the first year, a mean number of 8.3 (± 2.4) injections were administered to the study eye. This roughly corresponds to the injection intensities seen in other routine T&E IVT-AFL studies with observation periods of one year, as reported by Abdin et al.^19^ and Yamamoto et al.^20^. With a mean of 5.4 (± 2.9) injections during the second year, the number of applied IVT-AFL injections is slightly lower than reported in some other routine T&E IVT-AFL regimens^17,18^ but slightly higher than reported by Traine and co-workers^15^. However, no correlation between the number of IVT-AFL injections and BCVA outcomes was found in our study. In the T&E regimen, patients with low disease activity require less frequent treatment while typically obtaining good BCVA results. This lack of correlation may therefore suggest that there was probably no systematic undertreatment in this study, which is discussed as one of the main reasons for a gradual decline in visual acuity over time in routine anti-VEGF treatment^21^. Accordingly, there was only a slight increase in the mean time between IVT-AFL injections from 63.3 (± 22.0) days at months 12 to 69.1 (± 28.6) days at month 24. This may indicate that after one year of therapy there is only minimal change of the individual treatment interval, and relative stability can be reached within the first year.

At month 12, the observed treatment interval was ≥ 8 weeks for 65.2% of patients. 37.0% of patients even reached a treatment interval of at least 12 weeks at month 24. However, patients with premature cessation of IVT-AFL therapy due to the absence of disease activity were not considered in the observed cases analysis at 24 months. Even though individual criteria may have slightly varied among the study sites, the majority of patients with treatment suspension due to success had consecutive injections at intervals of at least 12 weeks or longer without any disease recurrence before treatment was suspended. On the other hand, 38% of patients required treatment more frequently than every 8 weeks after 24 months of treatment. This is more intense treatment than the standard treatment label allowed in most countries except Switzerland. This study convincingly corroborates that one fixed treatment paradigm does not fit all, and that a considerable number of patients need more frequent injections than every 8 weeks. The level of intraocular VEGF suppression need varies from patient to patient, which confirms the content of specific individually different VEGF suppression times for patients^6^. These findings are also important for future clinical nAMD studies, which should allow IVT-AFL treatment intervals of less than 8 weeks.

Besides the aforementioned aspects, it is interesting to compare our study results with those reported in the ALTAIR^22^ and ARIES^23^ studies with a focus on the baseline visual acuity and the geographic area of the study. In these studies, T&E regimens were used after a loading phase consisting of 3 monthly AFL injections and an 8-weekly interval. Whereas ALTAIR was conducted in Japan and compared 2-weekly and 4-weekly extension intervals, the ARIES study population was predominantly Caucasian and differences between an early switch to T&E and a late switch were investigated. The letter gain at 12 months in the ALTAIR study was 8.4–9.0 letters, similar to our results, but with fewer injections (6.9–7.2). This might be explained by the lower baseline visual acuity (55 ETDRS letters) and a higher proportion of PCV (37% vs 8%), which in our study had the highest gain in BCVA. In line with this observation, during the second year only 3.6 AFL injections were necessary to maintain the vision gain. The mean last treatment interval was almost 2 weeks longer in ALTAIR both at 12 and 24 months, and the proportion of patients with a last interval > 12 weeks was considerably higher (42–50% and 57–60% at 12 months and 24 months, respectively). Overall, the results in the ARIES study are more similar to our findings. The letter gain from baseline was 4.3–7.9 letters at 24 months, which is comparable to the 5.0 letters in our sample. Equally, the number of AFL injections over 104 weeks was 12–13 treatments, similar to the 13.7 injections reported here. The close replication of ARIES findings in our study might be due to similar baseline visual acuity (61 letters) and the common Caucasian background. Of note, in all these T&E studies, the maximal treatment interval does not change much anymore between 12 and 24 months, a finding also replicated in our study.

There are some limitations which are mainly due to the observational study design, prohibiting predefined study assessments as well as timing of visits, and the partly retrospective collection of data. On the other hand, these aspects prevented any type of influence on investigators´ decisions and helped to underpin the study´s real life setting.

In conclusion, the presented data show that a T&E regimen using AFL in Swiss routine practice resulted in anatomic and functional outcomes similar to those from prospective registration trials of AFL for nAMD treatment.

## Supplementary information

Supplementary Information.

## Data Availability

Due to the small sample size, datasets generated during the study are not publicly available in order to ensure that patient privacy is safeguarded but are available from the corresponding author on reasonable request and with permission of the study´s funder.

## References

[1] Gragoudas ES, Adamis AP, Cunningham ET, Feinsod M, Guyer DR, VEGF Inhibition Study in Ocular Neovascularization Clinical Trial Group (2004). Pegaptanib for neovascular age-related macular degeneration. N. Engl. J. Med..

[2] Rosenfeld PJ (2006). Ranibizumab for neovascular age-related macular degeneration. N. Engl. J. Med..

[3] Brown DM (2006). Ranibizumab versus verteporfin for neovascular age-related macular degeneration. N. Engl. J. Med..

[4] Holz FG (2015). Multi-country real-life experience of anti-vascular endothelial growth factor therapy for wet age-related macular degeneration. Br. J. Ophthalmol..

[5] Mantel I, Deli A, Iglesias K, Ambresin A (2013). Prospective study evaluating the predictability of need for treatment with intravitreal ranibizumab for age-related macular degeneration. Graefes Arch. Clin. Exp. Ophthalmol..

[6] Fauser S, Schwabecker V, Muether PS (2014). Suppression of intraocular vascular endothelial growth factor during aflibercept treatment of age-related macular degeneration. Am. J. Ophthalmol..

[7] Papadopoulos N (2012). Binding and neutralization of vascular endothelial growth factor (VEGF) and related ligands by VEGF Trap, ranibizumab and bevacizumab. Angiogenesis.

[8] Stewart MW, Rosenfeld PJ (2008). Predicted biological activity of intravitreal VEGF Trap. Br. J. Ophthalmol..

[9] Spaide R (2007). Ranibizumab according to need: a treatment for age-related macular degeneration. Am. J. Ophthalmol..

[10] Berg K, Pedersen TR, Sandvik L, Bragadóttir R (2015). Comparison of ranibizumab and bevacizumab for neovascular age-related macular degeneration according to LUCAS treat-and-extend protocol. Ophthalmology.

[11] Gregori NZ, Feuer W, Rosenfeld PJ (2010). Novel method for analyzing snellen visual acuity measurements. Retina.

[12] DeCroos FC (2017). Treat-and-extend therapy using aflibercept for neovascular age-related macular degeneration: a prospective clinical trial. Am. J. Ophthalmol..

[13] Eleftheriadou M (2018). Three-year outcomes of aflibercept treatment for neovascular age-related macular degeneration: evidence from a clinical setting. Ophthalmol. Ther..

[14] Talks JS (2016). First-year visual acuity outcomes of providing aflibercept according to the VIEW study protocol for age-related macular degeneration. Ophthalmology.

[15] Traine PG, Pfister IB, Zandi S, Spindler J, Garweg JG (2019). Long-term outcome of intravitreal aflibercept treatment for neovascular age-related macular degeneration using a "treat-and-extend" regimen. Ophthalmol. Retina.

[16] Heier JS (2012). Intravitreal aflibercept (VEGF trap-eye) in wet age-related macular degeneration. Ophthalmology.

[17] Barthelmes D (2018). Two year outcomes of „treat and extend“ intravitreal therapy using aflibercept preferentially for neovascular age-related macular degeneration. Retina.

[18] Jaki Mekjavić P, Gregorčič B, Oberč C, Podgoršek S (2018). Treat-and-extend therapy using intravitreal aflibercept for neovascular age-related macular degeneration: 2-year real-world practice data from Slovenia. BMC Ophthalmol..

[19] Abdin AD, Suffo S, Asi F, Langenbucher A, Seitz B (2019). Intravitreal ranibizumab versus aflibercept following treat and extend protocol for neovascular age-related macular degeneration. Graefes Arch. Clin. Exp. Ophthalmol..

[20] Yamamoto A, Okada AA, Nakayama M, Yoshida Y, Kobayashi H (2017). One-year outcomes of a treat-and-extend regimen of aflibercept for exudative age-related macular degeneration. Ophthalmologica.

[21] Monés J (2020). Undertreatment of neovascular age-related macular degeneration after 10 years of anti-vascular endothelial growth factor therapy in the real world: the need for a change of mindset. Ophthalmologica.

[22] Ohji M (2020). Efficacy and safety of intravitreal aflibercept treat-and-extend regimens in exudative age-related macular degeneration: 52- and 96-week findings from ALTAIR: a randomized controlled trial. Adv. Ther..

[23] Souied EH (2020). Efficacy of intravitreal aflibercept treat-and-extend regimen over 2 years for neovascular age-related macular degeneration: ARIES study. Invest. Ophthalmol. Vis. Sci..

