# Supplementary material for: Two-year outcomes of intravitreal aflibercept in a Swiss routine treat and extend regimen for patients with neovascular age-related macular degeneration
Source: Sci Rep. 2020 Nov 20;10:20256. doi: 10.1038/s41598-020-76354-1 (PMC7680100; doi:10.1038/s41598-020-76354-1)
Supplement: Supplementary file 1 — Supplementary Information. [file 41598_2020_76354_MOESM1_ESM.pdf]

Supplementary information

Two-year outcomes of intravitreal aflibercept in a Swiss routine treat and extend regimen for patients with neovascular age-related macular degeneration

Andreas Ebneter, Stephan Michels, Christian Prünte, Pascal Imesch, Felix Eilenberger, Susanne Oesch, Isabelle P. Thomet-Hunziker & Katja Hatz

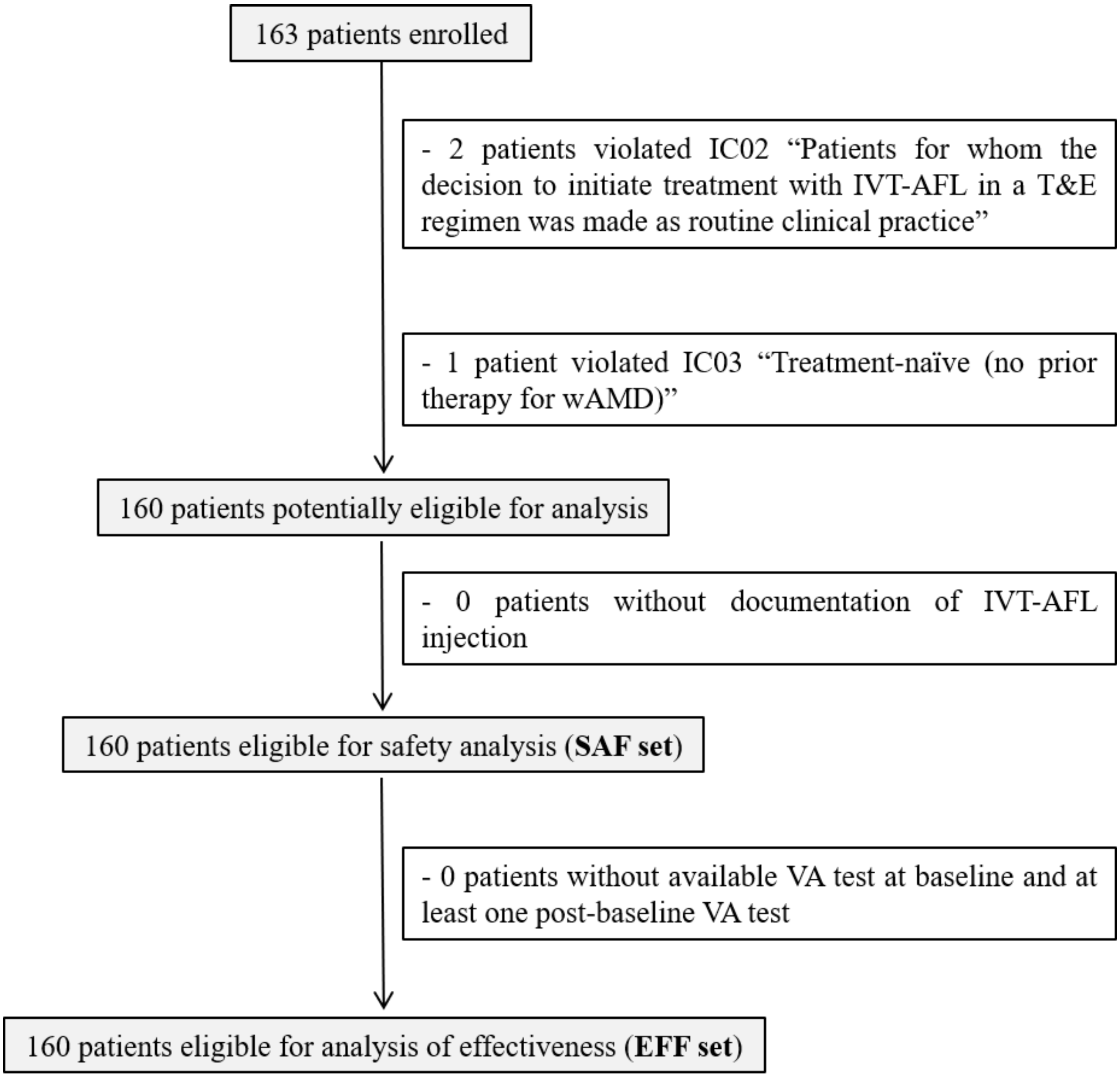

Supplementary Fig. S1: Patient distribution

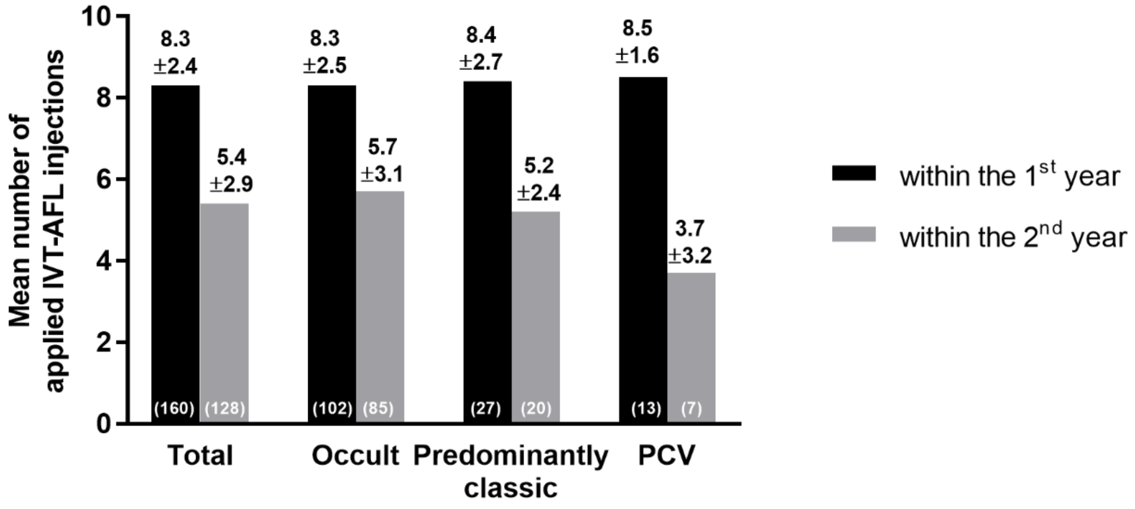

Supplementary Fig. S2: Number of applied IVT-AFL injections into the study eye within the 1st and the 2nd year after aflibercept treatment initiation for the total EFF population and the three main CNV type subgroups. The numbers above the columns indicate the mean ± standard deviation. The numbers in brackets given on the base of each column indicate the number of patients with available information.

Supplementary Table S1: Treatment-emergent adverse events (TEAE) according to MedDRA-SOC and PT (patient based)

TEAE (patient based)			*N*	*%*
Any SOC			17	10.63
Eye disorders	Any PT		12	7.50
		Cataract	1	0.63
		Corneal erosion	2	1.25
		Detachment of retinal pigment epithelium	1	0.63
		Erythema of eyelid	1	0.63
		Neovascular age-related macular degeneration	5	3.13
		Retinal degeneration	1	0.63
		Retinal detachment	1	0.63
		Subretinal fluid	1	0.63
Immune system disorders	Any PT		1	0.63
		Reaction to preservatives	1	0.63
Infections and infestations	Any PT		4	2.50
		Dacryocystitis	1	0.63
		Diverticulitis	1	0.63
		Ophthalmic herpes zoster	1	0.63
		Pneumonia	1	0.63
		Sepsis	1	0.63
Injury, poisoning and procedural complications	Any PT		2	1.25
		Hip fracture	1	0.63
		Traumatic haematoma	1	0.63
Investigations	Any PT		1	0.63
		Intraocular pressure increased	1	0.63
Nervous system disorders	Any PT		1	0.63
		Coma	1	0.63
Surgical and medical procedures	Any PT		4	2.50
		Cataract operation	4	2.50
Number of patients included in safety analysis			160	100.00
